# Transcription factor PAX4 facilitates gastric cancer progression through interacting with miR-27b-3p/Grb2 axis

**DOI:** 10.18632/aging.203214

**Published:** 2021-06-23

**Authors:** Yan Zhang, Li Ding, Qingfeng Ni, Ran Tao, Jun Qin

**Affiliations:** 1 Department of General Surgery, Affiliated Hospital of Nantong University, Nantong 226001, Jiangsu, PR China; 2 Department of Chemotherapy, Affiliated Hospital of Nantong University, Nantong 226001, Jiangsu, PR China

**Keywords:** gastric cancer, PAX4, miR-27b-3p, Grb2

## Abstract

Gastric cancer (GC) is one of the most common aggressive cancers. The discovery of an effective biomarker is necessary for GC diagnosis. In this study, we confirmed that Paired box gene 4 (PAX4) is up-regulated in GC tissues and cells via quantitative real time polymerase chain reaction (qRT-PCR), western blot and immunohistochemical staining. It was also identified that PAX4 contributed to GC cell proliferation, migration and invasion through Cell Counting Kit-8, BrdU, flow cytometry assay, colony formation assay, transwell assays, and wound healing assay. miR-27b-3p was confirmed with the binding site with PAX4 using ChIP assay and served as a tumor suppressor that inhibiting GC cell growth and metastasis, and reversed the effect of PAX4. Bioinformatics prediction and dual luciferase assay results demonstrated that miR-27b-3p targeted Grb2, which could alter the function of miR-27b-3p. Furthermore, the transcriptional control of PAX4-regulated miR-27b-3p activated the Ras-ERK pathway. Taken together, the PAX4/miR-27b-3p/Grb2 loop is known to be involved in GC cell promotion, and can be seen as a promising target for GC therapy.

## INTRODUCTION

According to the global cancer statistics released by the International Agency for Research on Cancer in 2018, gastric cancer (GC) is one of the most common cancers and is associated with limited effective treatments for the disease [1, 2]. Although chemotherapy can prolong the survival time of a few patients with advanced GC, it is characterized by high morbidity and mortality due to short maintenance time and poor effectiveness of the majority of chemotherapy methods [3]. Gastroscopic screening studies in South Korea and Japan have demonstrated that GC screening is one of the effective strategies for reducing GC-associated mortality [4]. The reason why this strategy is effective is that population screening can help identify GC as early as possible. Using GC biomarkers with good clinical results for detection, instead of gastroscopic screening, has been recognized widely [5] and can help achieve a higher popularization rate, early detection, early diagnosis and early treatment. Biomarkers are the biochemical indicators that can mark changes or possible changes in the structure or function of systems, organs, tissues, cells and subcellular compartments. Previous studies have identified potential GC markers, such as carbohydrate antigen (CA) and carcinoembryonic antigen (CEA) [6], PD-L1 and APE1 [7], miRNAs [8], epidermal growth factor receptor (HER) family [9], and fibroblast growth factor (FGFRs), for clinical application [10].

Paired box 4 (PAX4), a member of the paired homologous domain transcription factor PAX gene family, is located on chromosome 7q32 [11]. The DNA binding domain of PAX4 is formed by a homeo-like domain, as well as the paired structural domain, and belongs to the fourth subfamily of the PAX family [12, 13]. PAX family transcription factors take on essential functions in tissue development and cell differentiation. For example, PAX4 is involved in regulating pancreatic β-cell proliferation and survival in diabetes [14–16]. PAX4 displays both immune and regenerative properties, and combination with downstream targets offers a new prospective in diabetes treatment. This is aimed at preserving the remaining β-cells in parallel to stimulating their proliferation in order to replenish the mass of β-cells lost during disease progression [17, 18]. Studies over the past decades have demonstrated that PAX genes are abnormally expressed in human malignant medulloblastoma, ovarian cancer and thyroid cancer [19–22], which can help promote proliferation and inhibit apoptosis of cancer cells via mitosis and regulation of target genes or miRNAs. It has also been reported that PAX4 corresponds to aberrant DNA demethylation in the promoter region, and acts as a candidate oncogene in hematologic malignancies [23]. PAX4 is redundant for proliferation of insulinoma cells by upregulating the antiapoptotic gene bcl-xl. [24] Although the relationship between PAX4 and diabetes or tumors has gained significant attention, the performance of PAX4 in GC has not yet been reported and the regulatory mechanism of PAX4 expression remains elusive.

MiRNAs are a class of short (20–23nt) endogenous single-stranded RNA molecules that are encoded by RNA genes in the non-protein coding region of the eukaryotic genome, which regulate target gene expression at the post-transcriptional level. There is firm evidence that aberrant miRNAs expression is associated with tumorigenesis. MiR-27b-3p was found at low levels in lung cancer cells, and inhibits cancer cells transition and serves as a tumor suppressor [25]. Growth factor receptor-bound protein 2, Grb2, is an essential adaptor protein that is widely expressed in cells [26]. In addition, Grb2 acts as a major signal transduction molecule that plays a role in multiple downstream cancer signaling pathways. It has been reported that elevated expression of Grb2 was identified in various cancer cells, including breast cancer, GC and esophageal squamous cancer [27–29]. MiRNA and its downstream target are hotspots in the lncRNA interaction discovery. Herein, we build a hypothesis based on experimental results that PAX4 is up-regulated and is closely associated with GC. We applied bioinformatics methods and dual luciferase assay in order to investigate that PAX4 binds to the corresponding downstream DNA promoters in order to inhibit miR-27b-3p transcription, and further up-regulates Grb2 levels, accompanied by Ras-ERK pathway activation, and therefore contributes to GC cells promotion.

## MATERIALS AND METHODS

### Patient tissues

Overall, 60 GC patients that were admitted to the Affiliated Hospital of Nantong University were selected between January 2015 to December 2015 to participate in the study. This study was granted approval by the hospital ethics committee, and all patients were given informed consent. The following comprised the inclusion criteria: (1) patients must be diagnosed with GC with relevant clinical diagnosis; (2) patients must not have receives any anti-tumor treatment prior to surgery. The exclusion criteria stated that patients with organic diseases and other tumors were excluded. The tumor tissues and matched adjacent tissues of all patients were freshly collected and placed in a cryotube and immediately stored in liquid nitrogen.

### Construction of Pax4 and Grb2 over-expression vector

The Pax4 and Grb2 open reading frame (ORF) were amplified through the human cDNA library (Genbank: NM_001366110.1 and NM_173689.7) utilizing PrimeStar PCR and constructed into the expression vector CD513B in order to generate the CD513B-Pax4 ov plasmid and CD513B-Grb2 ov plasmid. The construct was validated via sequencing.

### Cell culture and transfection

Overall, six GC cell lines (HGC-27, MGC803, BGC-823, SGC-7901, AGS, MKN45) and the human gastric mucosal epithelial cell line GES-1, were cultured in RPIM 1640 complete culture medium containing 10% fetal bovine serum and 1% double antibodies (100 μg/mL penicillin and 100 μg/mL streptomycin) in an incubator containing 5% CO_2_ at 37°C.

Cells in the logarithmic growth phase were seeded into a six-well plates in complete culture medium. According to the instructions, once the cells grew to 50%–60% confluence degree, si-PAX4 and si-NC was transfected into AGS cells while PAX4 ov and NC were transfected into HGC-27 cells through the use of Lipofectamine™ ^3000^, respectively. MiR-27b-3p inhibitor/mimics (RIBOBIO) and Grb2 ov were further transfected into AGS cells. Next, qRT-PCR was utilized to determine the transfection efficiency for follow-up experiments.

### Quantitative real-time PCR (qRT-PCR)

Total RNA was extracted from tissue and cultured cells through the use of the TRizol reagent, following the guidelines, and then reverse transcribed into cDNA through the use of the Prime ScriptTM RT reagent Kit. The resulting cDNA was stored at –20°C for qPCR analysis. The expression of Pax4 and Grb2 was detected by ABI 7900HT RealTime PCR System using SYBR Green assays and GAPDH was the internal control. The expression of miR-27b-3p was measured using TaqMan MicroRNA Assays, with U6 as the internal control. The relative expression of PAX4 mRNA, Grb2 mRNA and miR-27b-3p were calculated via the RT-qPCR relative quantitative method (2^-ΔΔCt^ method). Each experiment was performed in triplicate.

### Transwell assays for cell migration and invasion

We added 200 μL of diluted matrigel to the upper chamber of the Boyden chamber assay and let it dry overnight. The transfected AGS cells and HGC-27 cells were put into a single cell suspension and then rinsed with serum-free medium three times. After cell counting, the cells were seeded onto the upper chamber of the transwell chamber, and 300 μL DMEM medium, containing 10% FBS, which served as a chemokine, was added to the lower chamber. The plates were added to a 37°C and 5% CO_2_ incubator. Next, the plates were taken out the chamber after 24 h, and soaked and washed three times in PBS. Then, the cells in the transwell upper chamber were fixed in 95% ethanol, washed three times with PBS, soaked with freshly prepared crystal violet staining solution for 5–10 minutes, and a cotton swab was used to wipe the cells in the upper chamber that had not yet gone through the filter membrane. The cells were observed under the microscope, pictures were taken, and cells were counted. Then, the average value was taken in order to evaluate the invasion ability by assessing the number of cells that migrated to the lower layer of the microporous membrane. For the migration assay, we left out the step of treating the upper chamber with Matrigel. The rest of the procedures were the same. Each experiment was performed in triplicate.

### Cell proliferation assays

Cell proliferation was evaluated using the CCK-8 assay. GC cell lines (AGS and HGC-27), 24 h after transfection, were seeded onto the 96-well plate at a density of 4000 cells per well. Transfected no-carrier cells were used as the control group (Vector). There were three duplicate wells were set for each treatment. Cells were cultured for 24, 48, 72 and 96 h. Next, 10 μL of the CCK-8 solution was added to each well and incubated at 37°C for 1 h. The optical density (OD) value at 450 nm was measured using a micrometer. All experiments were repeated three times.

### BrdU assay

The cells were seeded onto a 96-well plate. Next, 24 h after transfection, cell proliferation was determined by the BrdU ELISA kit. The operation procedure was carried out according to instructions. First, 15 μL BrdU solution was added to each well, and incubated at 37°C, 5% CO_2_ for 2 h. Next, the supernatant was replaced with 100 μL denaturated solution and cells were incubated for 10 min. According to instruction, the BrdU antibody, secondary antibody and tetramethylbenzidine were successively added for incubation for 30 min. Next, the absorbance at 370 nm was measured through a microplate reader in order to reflect the changes in cell proliferation. Finally, we prepared three multiple holes for each set of samples; the experiments were repeated three times.

### Flow cytometry assay

GC cells (AGS and HGC-27) within each group were diluted and inoculated into a six-well plate with 1 × 10^4^ cells per well, and cultured for 72 h. Then, the cells were digested with trypsin and the cell suspension was collected. Annexin V-FITC (5 μL) and propidium iodide (5 μL) were mixed according to the instructions of the apoptosis kit. The apoptosis rate was detected and analyzed using the flow cytometer. The experiment was repeated three times.

### Western blot

The transfected cells were collected after 24 h, and the cells were lysed using RIPA lysis buffer in order to extract total protein. The protein concentration was determined using the BCA method. Proteins were then separated by 10% SDS-PAGE gel electrophoresis, and transferred to a membrane, blocked with 5% skimmed milk powder at room temperature for 1 h. The membranes were then incubated with primary antibodies targeting Pax4 (PA1-108, ThermoFisher), Grb2 (ab32111; Abcam), p-Raf (ab157201; Abcam), T-Raf (ab230850; Abcam), p-MEK (ab278562; Abcam), t-MEK (ab32576; Abcam), p-ERK (ab136926; Abcam), t-ERK (ab184699; Abcam), Vimentin (ab92547; Abcam), Bax (ab32503; Abcam), CyclinD1 (ab16663; Abcam) and GAPDH (ab181602; Abcam). The membranes were incubated at 4°C overnight, washed with TBST three times, five min each time. Then, the membranes were incubated with secondary antibodies for 1 h at room temperature. The membranes were washed with TBST three times, five min each time. Finally, ECL luminescent solution was added to the dark room, exposed and developed.

### Wound healing assay

Transfected AGS and HGC-27 cells were seeded onto a six-well plate at 1 × 10^6^/well, and cultured to 80% confluency. The monolayer of cells was scarped with a 200 μL pipette tip, and then the cells were continued to be cultured in FBS-free medium. The wound healing was observed under an inverted microscope, with photographs taken at 0 and 48 h. The wound healing rate was determined by measuring the wound width using the ImageJ software. The calculation is as follows: wound healing rate = (wound width-wound width at different time points/original wound width × 100%). Each experiment was performed in triplicate.

### Colony formation assay

In order to carry out the colony formation assay, 24 h after transfection, cells were digested and evenly seeded onto a 6-well plate at a density of 5000 cells per well. The cells were divided into groups, and added into multiple wells. The clones were cultured in an incubator for approximately 14 days to observe the number of colonies. At the same time, the clones were visualized and counted. The experiment was repeated three times.

### Chromatin immunoprecipitation assay

Chromatin immunoprecipitation (ChIP) assays were carried out utilizing an ImprintH Chromatin Immunoprecipitation Kit Sigma (St. Louis, MO), based on protocol provided by the manufacturer. Briefly, cells were cross-linked using 1% formaldehyde, which was followed by nuclear fractionation and DNA shearing via sonication. The anti-HA antibody (ab9110; Abcam) was utilized to perform immunoprecipitation and rodent immunoglobulin G (IgG) was used as negative control. Following extensive washing, the bound DNA fragments were eluted and analyzed by PCR. The primer sequences are listed in Table 1.

**Table 1:** The primers used in this study.

**Name**	**Sequences**
Pax4 forward primer	5′-ATACCCGGCAGCAGATTGTG-3′
Pax4 reverse primer	5′-AAGACACCTGTGCGGTAGTAA-3′
miR-27b-3p forward primer	5′-AGCGTTCACAGTGGCTAAG-3′
miR-27b-3p reverse primer	5′-TCCTCCTCTCCTCTCCTCTC-3′
Crb2 forward primer	5′-ACCACTGTGCTTGTCCTGAG-3′
Crb2 reverse primer	5′-TCCAGGGTCGCTAGATGGAG-3′
U6 forward primer	5′-CTCGCTTCGGCAGCACA-3′
U6 reverse primer	5′-GGATGGTGATGGTTTGGTAG-3′
GAPDH forward primer	5′-TCCTCTGACTTCAACAGCGACAC-3′
GAPDH reverse primer	5′-CACCCTGTTGCTGTAGCCAAATTC-3′
P1 forward primer (Chip)	5′-CCCCTGGCTTCTATTGGAGT-3′
P1 reverse primer (Chip)	5′- GTGTAGAAACCCATACTGAG-3′
P2 forward primer (Chip)	5′-CTGTGTCTGCTCCGTTTTTC-3′
P2 reverse primer (Chip)	5′-AACACCAAATATTCAATAT-3′
P3 forward primer (Chip)	5′-TCACTCAGTTTTGAACATTGA-3′
P3 reverse primer (Chip)	5′- CCTGACTCTTCTGGAAGAATA-3′

### Dual luciferase assay

The transfected AGS cells were collected and seeded onto a 24-well plate (1 × 10^4^ cells per well). The cells were cultured and observed to determine whether the grew into one layer and constructed the wild-type (pGL3-basic-miR-27b-3p promoter-wt) and mutant (pGL3-basic-miR-27b-3p promoter-mut) dual luciferase reporter vector, co-transfected with NC or PAX4 ov, respectively. Simultaneously, we applied the wild-type (pGL3-control-Grb2 3p′UTR-wt) and mutant (pGL3-control-Grb2 3p′UTR-mut) dual luciferase reporter vectors in order to transfect miR-NC or miR-27b-3p, respectively. Then, 48 h after transfection, the RIPA lysate was lysed at room temperature for 20 minutes, after which the supernatant was gathered via centrifugation. The luciferase substrate was then added and activity was determined by the photoluminescence instrument.

### Immunohistochemical staining (IHC)

All tissues were paraffin-embedded and acquired from the Department of Pathology at the Affiliated Hospital of Nantong University. The paraffin-embedded tissues were sliced into 4-μm sections. The tissues slices were dewaxed, dehydrated and rehydrated. A rabbit anti-Pax4 polyclonal antibody (PA1-108, ThermoFisher) was added and incubated into sections overnight at 4°C. The SP-9000 HistostainTM Plus kits (ZSGB-BIO) were utilized as per the manufacturer’s protocol. Tumor slices were then evaluated in a blinded manner. Ten fields were chosen for examination of cell-staining intensity and proportion of positive cells. Immunohistochemical staining was evaluated according to the immunoreactive score (IRS), and then evaluated using the staining intensity and proportion of positive cells. The staining intensity was graded as 0 (no staining), 1 (weak), 2 (moderate) and 3 (strong). The proportion of positive cells were scored as 0 (negative), 1 (<10%), 2 (10–50%) or 3 (>50%). Both scores were multiplied and the IRS was determined. Values ≥3 were defined as positive cytoplasmic expression, and values <3 were regarded as negative.

### Animal experiments

Five-week-old BALB/C nude mice were acclimated to room temperature routinely. The mice were divided into four groups, and subcutaneously injected with AGS and HGC-27 cells that were transfected with si-NC, si-PAX4 or NC, and PAX4 ov into mice, respectively. Tumor growth was observed every three days until three weeks post-injection. The mice were then handled, and the tumor weight was measured. The calculation for tumor volume was as follows: tumor volume (mm^3^) = 0.5× length (mm) × width (mm) × height (mm). All experiments abided by protocols set by the Animal Ethics Committee of local hospital.

### Statistical analysis

Graph Pad Prism 5 software and SPSS 22.0 were utilized for statistical analyses. The data is expressed as mean ± standard deviation. The independent sample *t* test was used to compare two groups, while one-way analysis of variance was used for the comparison between multiple groups. Pearson’s coefficient correlation or linear regression analysis helped determine the relationship between two variables. Categorical data were assessed using a chi-square test. Survival rates were determined via the Kaplan-Meier method. A log-rank test helped compare significance. *P* < 0.05 refers to a statistically significant difference.

## RESULTS

### GC tissues and cells have elevated PAX4 mRNA and protein levels

**Figure 1:**
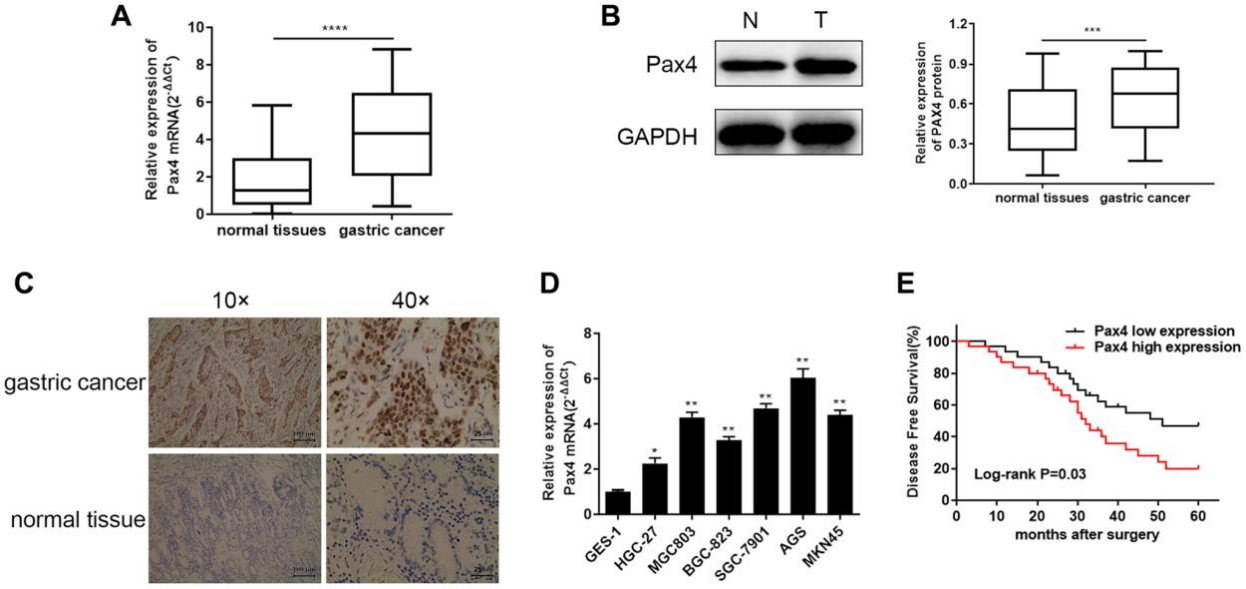
**Elevated PAX4 mRNA and protein expression in both GC tissues and cell lines.** (**A**, **B**) Higher PAX4 mRNA and protein expression in GC tissues compared to normal tissues (*n* = 60) by qRT-PCR (*P* < 0.0001) and WB (*P* = 0.0002). (**C**) The IHC results between GC and non-GC tissues demonstrated excessive PAX4 in GC tissues. (**D**) Overexpression of PAX4 mRNA levels were identified across six GC cell lines (HGC-27, MGC803, BGC-823, SGC-7901, AGS, MKN45) compared to the human gastric mucosal epithelial cell line GES-1. (**E**) The overall survival analysis results demonstrated that PAX4 high expression in GC is associated with poor prognosis.

In order to determine the differential PAX4 expression in GC patients, we examined PAX4 mRNA and protein expression across 60 pairs of GC tissues and adjacent normal tissues using qRT-PCR, WB and IHC. The differentially significant PAX4 mRNA and protein levels are displayed in Figure 1A–1C between GC tissues and paired adjacent normal tissues. The results indicated that PAX4 is overexpressed in GC patients’ tissues compared to non-GC tissues. A similar trend was observed across six GC cell lines (HGC-27, MGC803, BGC-823, SGC-7901, AGS, MKN45), compared to the human gastric mucosal epithelial cell line GES-1. Among the six GC cell lines, the AGC cell line demonstrated the highest PAX4 expression while HGC-27 cell line had relatively low PAX4 mRNA levels (Figure 1D). As observed in the overall survival analysis, elevated PAX4 expression was related to a lower 5-year survival and poor prognosis in GC patients (Figure 1E). Furthermore, we studied the relationship between Pax4 expression and the clinic-pathologic features of GC patients. As shown in Table 2, Pax4 expression was significantly correlated to tumor size (*P* = 0.0371), lymph node metastasis (*P* = 0.0029) and TNM stage (*P* = 0.0039). However, it was not related to age, gender, or histological grade.

**Table 2:** Correlation of Pax4 expression with clinicopathological characteristics in GC patients.

**Characteristic**	**Case**	**Pax4 expression**		***P* value**
		**Low**	**High**	
All case	60	30	30	
Age (years)				0.432
<60	25	11	14	
≥60	35	19	16
Gender				0.292
Female	24	14	10	
Male	36	16	20
Tumor size (cm)				***0.0371***
<3cm	26	17	9	
≥3cm	34	13	21
Histological grade				0.063
High	37	22	15	
Low-middle	23	8	15
Lymph node metastasis				***0.0029***
Negative	39	25	14	
Positive	21	5	16
TNM stage				***0.0039***
I-II	25	18	7	
III-IV	35	12	23

### PAX4 facilitated GC cell proliferation, migration and invasion, inhibited cancer cell apoptosis and promoted tumor growth

**Figure 2:**
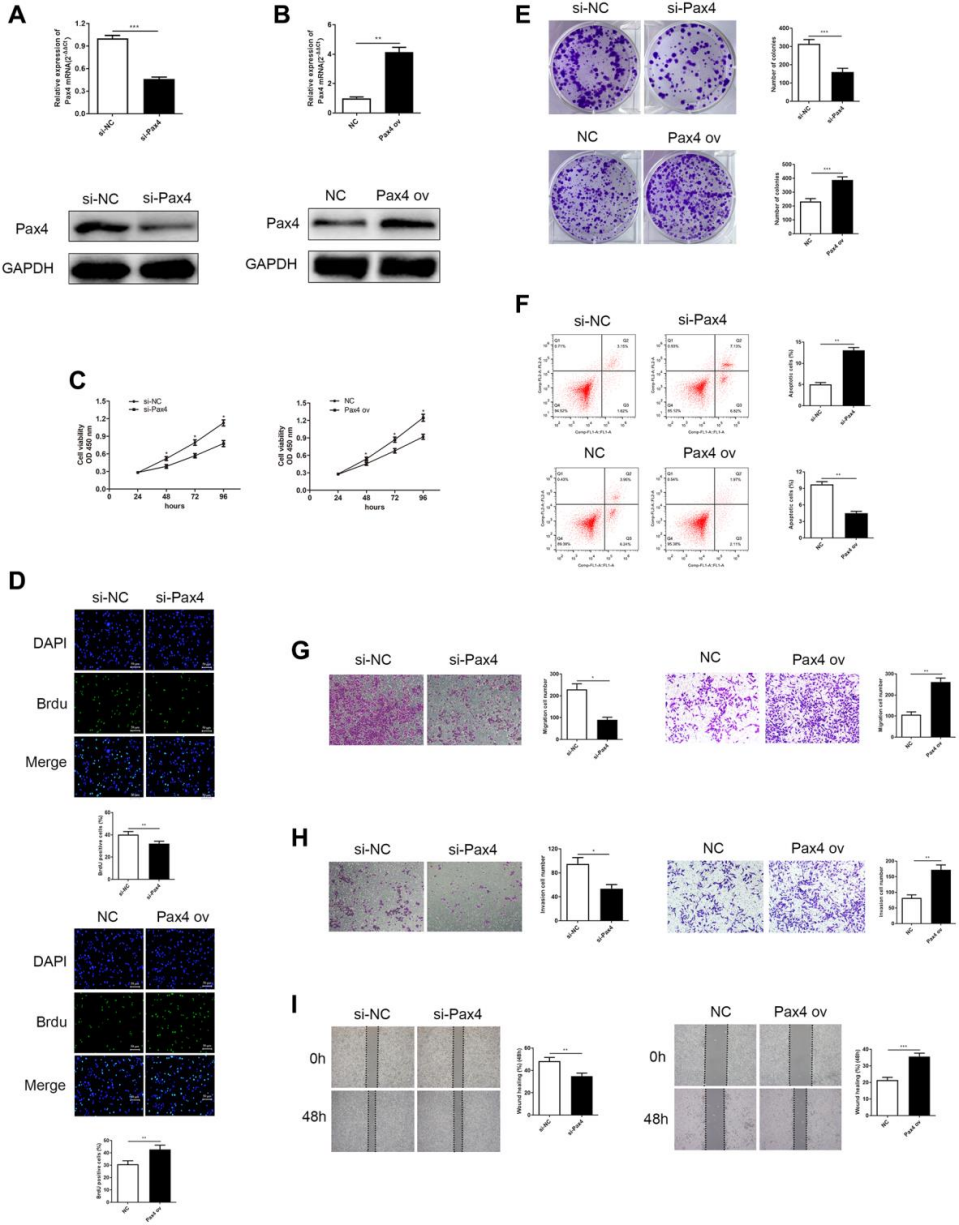
**Knockdown of PAX4 in AGS cells inhibited GC cell proliferation, migration and invasion, while overexpression of PAX4 in the HGC-27 cells accelerates GC proliferation, migration and invasion.** (**A**) PAX4 mRNA and protein levels were decreased after si-PAX4 transfection in AGS cells compared to the control group (*P* = 0.0004). (**B**) PAX4 mRNA and protein levels were increased after PAX4 ov transfection in HGC-27 cells compared to the NC group (*P* = 0.0058). (**C**) Lower PAX4 expression weaken GC cell viability and higher PAX4 expression was beneficial for GC cell viability assessed by the CCK-8 assay. (**D**) Lower PAX4 expression weaken GC cell proliferation (*P* = 0.0016) and higher PAX4 expression was beneficial for GC cell proliferation (*P* = 0.0091) assessed by the Brdu assay. (**E**) Colony formation assay determined numbers of colony formation after si-PAX4 (*P* = 0.0005) or PAX4 ov (*P* = 0.0005) transfected (*P* = 0.0005). (**F**) Flow cytometry assay demonstrated that GC cell apoptosis capacity was strengthened in the absence of PAX4 (*P* = 0.0014) and was attenuated in PAX4 ov group (*P* = 0.0011). (**G**–**H**) Transwell assay demonstrated that decreased PAX4 result in inhibition of AGS cell migration (*P* = 0.012) and invasion (*P* = 0.013), increased PAX4 enhanced GC cell migration (*P* = 0.0014) and invasion (*P* = 0.0041). (**I**) Wound healing assay indicated the impaired GC cell migration when reduced PAX4 (*P* = 0.0076) and fortified GC cell migration when PAX4 was overexpressed (*P* = 0.0005).

As we previously mentioned, PAX4 overexpression is closely related to poor GC prognosis. Therefore, we hypothesized that PAX4 expression in GC cells may influence cancer cell progression. Therefore, we developed two systems to evaluate loss/gain of PAX4 function in GC cell life cycle. AGS cells were transfected with si-PAX4 and that helped conduct PAX4 knockdown, while HGC-27 cells were transfected with PAX4 ov aimed to build PAX4 overexpression. The corresponding control groups were transfected with si-NC and NC vectors, respectively. The PAX4 mRNA and protein expression were lower level when they were given si-PAX4 in AGS cells and had higher expression of PAX4 ov group of HGC-27 cells (Figure 2A and 2B).

Cell proliferation ability was measured using the CCK-8, BrdU and colony formation assays. A lack of PAX4 resulted in weaker GC cell growth, while excessive PAX4 levels promoted GC cell growth (Figure 2C–2E). Flow cytometry was utilized to determine cell apoptosis. Results indicated that knockdown of PAX4 could significantly increase GC cell apoptosis and overexpression of PAX4 inhibited apoptosis in HGC-27 cells (Figure 2F). Subsequently, transwell and wound healing assays measured cell migration and invasion. According to our results, AGS cells that lacked PAX4 expression presented worse migration and invasion abilities compared to the si-NC group, additionally, the migration and invasion abilities of HGC-27 cells with overexpressed PAX4 were distinctly stimulated, compared to the NC group (Figure 2G–2I).

**Figure 3:**
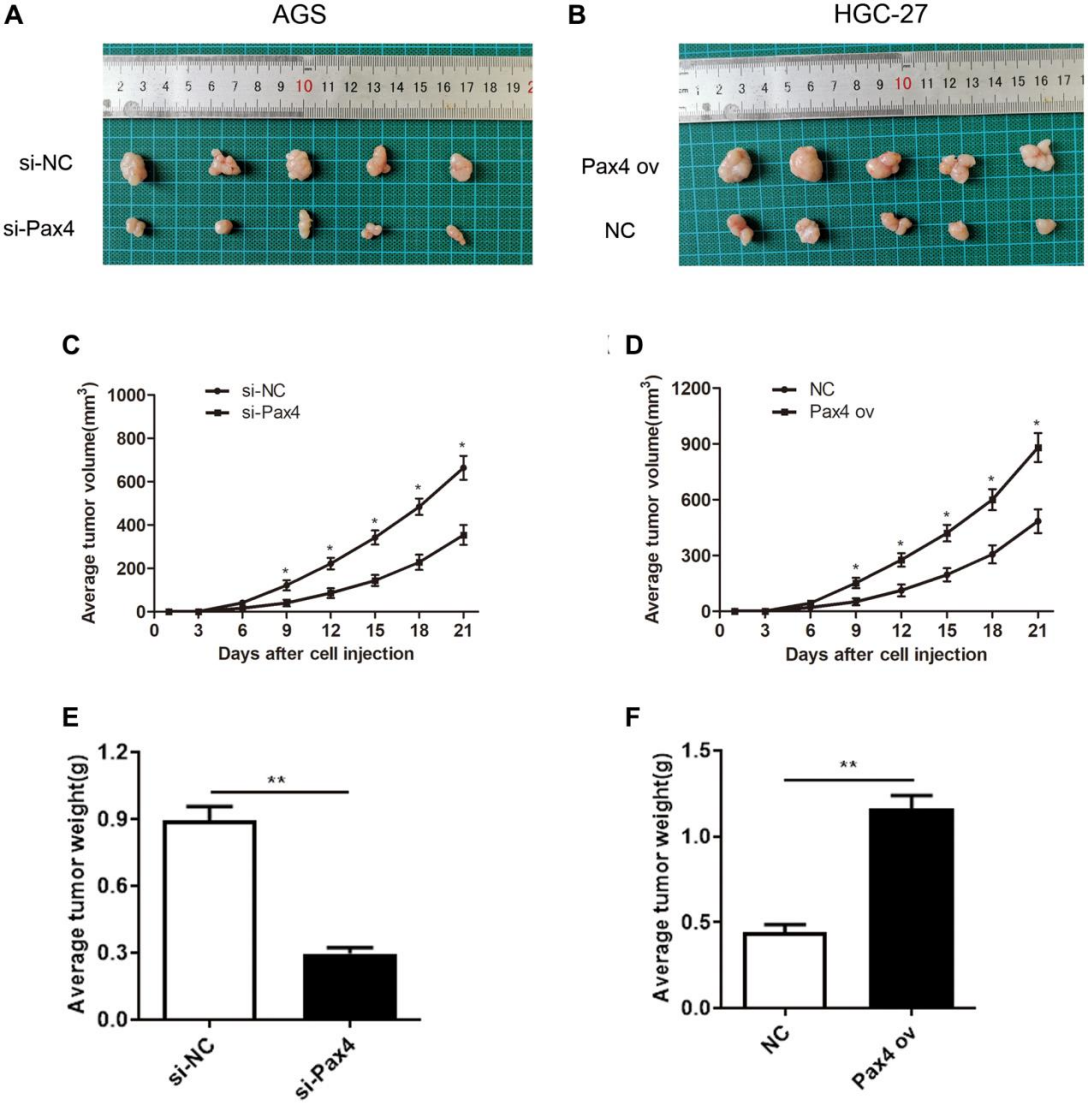
**PAX4 overexpression promoted GC tumor growth *in vivo*.** (**A**–**B**) Subcutaneous tumor records of the si-PAX4 group and PAX4 ov group. (**C**–**D**) The average tumor volume curves indicated that silencing PAX4 can inhibit tumor growth and upregulation of PAX4 can promote tumor growth. (**E**–**F**) Knockdown of PAX4 led to the lighter average tumor weight (*P* = 0.0039) and upregulating PAX4 led to an average heavier tumor weight (*P* = 0.0017).

In order to determine the function of PAX4 *in vivo*, we subcutaneously injected AGS cells that were transfected with si-PAX4 or si-NC and HGC-27 cells transfected with PAX ov or NC into nude mice, respectively. The records of tumor formation in the mice were observed and displayed (Figure 3A, 3B). Next, 21 days after injection, we observed that knockdown of PAX4 suppressed tumor growth. In fact, the average volume and weight of tumor in the si-PAX4 group were markedly decreased compared to si-NC group (Figure 3C, 3E). However, in the PAX4 ov group, the average volume and weight of tumor were significantly increased compared to the NC group (Figure 3D, 3F). Taken together, we concluded that up-regulated PAX4 levels can accelerate GC cell growth, migration and invasion, and promote GC tumor growth, functioned as a crucial oncogene both *in vitro* and *in vivo*.

### PAX4 targeted directly to miR-27b-3p and negatively regulated its expression

**Figure 4:**
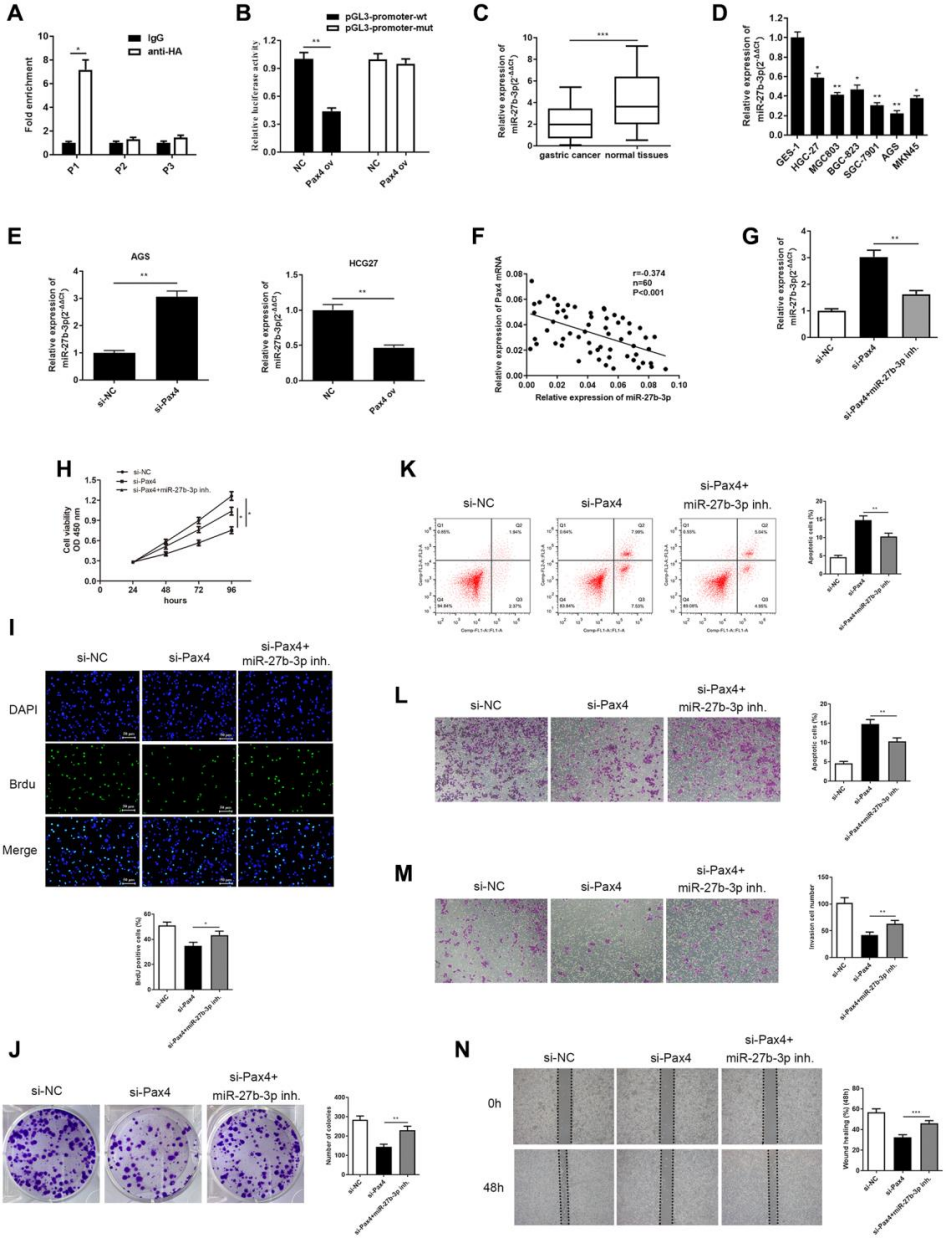
**PAX4 targets and negatively regulates miR-27b-3p, and miR-27b-3p reversed PAX4 function in AGS cells.** (**A**, **B**) The interaction between PAX4 and miR-27b-3p promoter region was validated through ChIP (*P* = 0.014) and dual luciferase assays (*P* = 0.0012). (**C**) The expression of miR-27b-3p was down-regulated in GC tissues (*n* = 60, *P* = 0.0004). (**D**) The expression of miR-27b-3p was down-regulated in six GC cell lines (HGC-27, MGC803, BGC-823, SGC-7901, AGS, MKN45) compared to the human gastric mucosal epithelial cell line GES-1. (**E**) Up-regulation of miR-27b-3p level was detected in the PAX4 knockdown group in AGS cells (*P* = 0.0061) and down-regulated miR-27b-3p levels were detected in the PAX4 overexpression group in HGC-27 cells by qRT-PCR (*P* = 0.0095). (**F**) There is a negative correlation between PAX4 and miR-27b-3p expression. (**G**) Lower miR-27b-3p levels were detected via qRT-PCR after addition of miR-27b-3p inhibitor to the si-PAX4 group (*P* = 0.0077). (**H**–**I**) CCK-8 and BrdU assays (*P* = 0.0035) detected that decreased miR-27b-3p level promoted enhanced GC cell viability and proliferation abilities. (**J**) Colony formation assay determined the numbers of colony formation (*P* = 0.0059). (**K**) GC cell apoptosis was examined via flow cytometry assay (*P* = 0.0077). (**L**–**M**) The increased cell migration (*P* = 0.0051) and invasion (*P* = 0.0051) abilities were identified when miR-27b-3p expression was inhibited by transwell assays. (**N**) Wound healing assay was utilized to discover enhanced GC cell metastasis in the absence of miR-27b-3p compared to the si-PAX4 group (*P* = 0.0006).

In order to fully discover the mechanism of PAX4 interaction in GC development, we determined the PAX4 target genes. Three Pax4 binding sites (P1: 459 to 466 bp; P2: 653 to 660 bp; P3: 829 to 836) were predicted, within the miR-27b-3p promoter region, using an online software (http://jaspar.genereg.net/). It was verified using ChIP assays that the activity data of Pax4 binding to the miR-27b-3p gene promoter was dramatically increased at site 1 (Figure 4A). Thus, we speculated that PAX4 may target miR-27b-3p in gastric cancer cells. To further validate this hypothesis, we constructed luciferase-reporter plasmids containing the miR-27b wild type promoter or mutant segments. In GC cells transfected with pGL3-miR-27b-3p promoter-wt plasmid, the relative luciferase activity of PAX4 ov group was significantly decreased compared to the NC group. Conversely, the relative luciferase activity of the PAX ov group transfected with pGL3-miR-27b-3p promoter-mut plasmid was not significantly different compared to the negative control group (Figure 4B). Furthermore, qRT-PCR was performed to determine expression of miR-27b-3p among GC tissues and cell lines. The results provided demonstrate that miR-27b-3p levels are relatively absent in GC tissues compared to normal tissues among 60 GC patients (Figure 4C). Furthermore, miR-27b-3p levels were down-regulated in six GC cell lines (HGC-27, MGC803, BGC-823, SGC-7901, AGS, MKN45) compared to the human gastric mucosal epithelial cell line GES-1 (Figure 4D). In AGS cells transfected with si-PAX4, miR-27b-3p was found to be overexpressed in the PAX4 knockdown group, compared to the control group, miR-27b-3p level was suppressed in HCG-27 cells with PAX4 overexpression (Figure 4E). Furthermore, Pearson’s correlation analysis revealed a negative relationship between PAX4 and miR-27b-3p expression (Figure 4F). These findings suggest that PAX4 directly targets miR-27b-3p and negatively regulates miR-27b-3p expression.

### MiR-27b-3p reversed PAX4-induced GC cells promotion and metastasis

In prior studies, we confirmed that PAX4 knockdown is able to restrain GC cell growth and metastasis. Furthermore, PAX4 acts as a transcriptional suppressor to inhibit miR-27b-3p levels. Hence, we speculate that PAX4 can exert its function by regulating miR-27b-3p. To validate this hypothesis, we conducted qRT-PCR to examine miR-27b-3p expression mediated by si-PAX4, si-PAX4+miR-27b-3p inhibitor and negative control in AGS cells. The levels of miR-27b-3p in the si-PAX4 group was highest, while adding miR-27b-3p inhibitor reduced miR-27b-3p expression (Figure 4G).

Furthermore, the corresponding rescue experiments were evaluated. CCK-8, BrdU and colony formation assays were carried out to evaluate cellular proliferation. These results demonstrated that silencing PAX4 is able to repress GC cells proliferation, while the presence of the miR-27b-3p inhibitor impairs this inhibition of cell growth (Figure 4H–4J). Additionally, we carried out flow cytometry to evaluate cell apoptosis, results of which indicated that adding the miR-27b-3p inhibitor rescued GC cell apoptosis brought by PAX4 knockdown (Figure 4K). Through transwell and wound healing assay, we determined that miR-27b-3p inhibitor reserved the inhibition of GC cell migration and invasion, as mediated by absence of PAX4 (Figure 4L–4N). Hence, these findings validate our hypothesis that miR-27b-3p is able to reverse GC cell promotion and metastasis mediated by PAX4 and can serve as a tumor suppressor.

### Grb2 targeted by miR-27b-3p and had a negative correlation with miR-27b-3p

**Figure 5:**
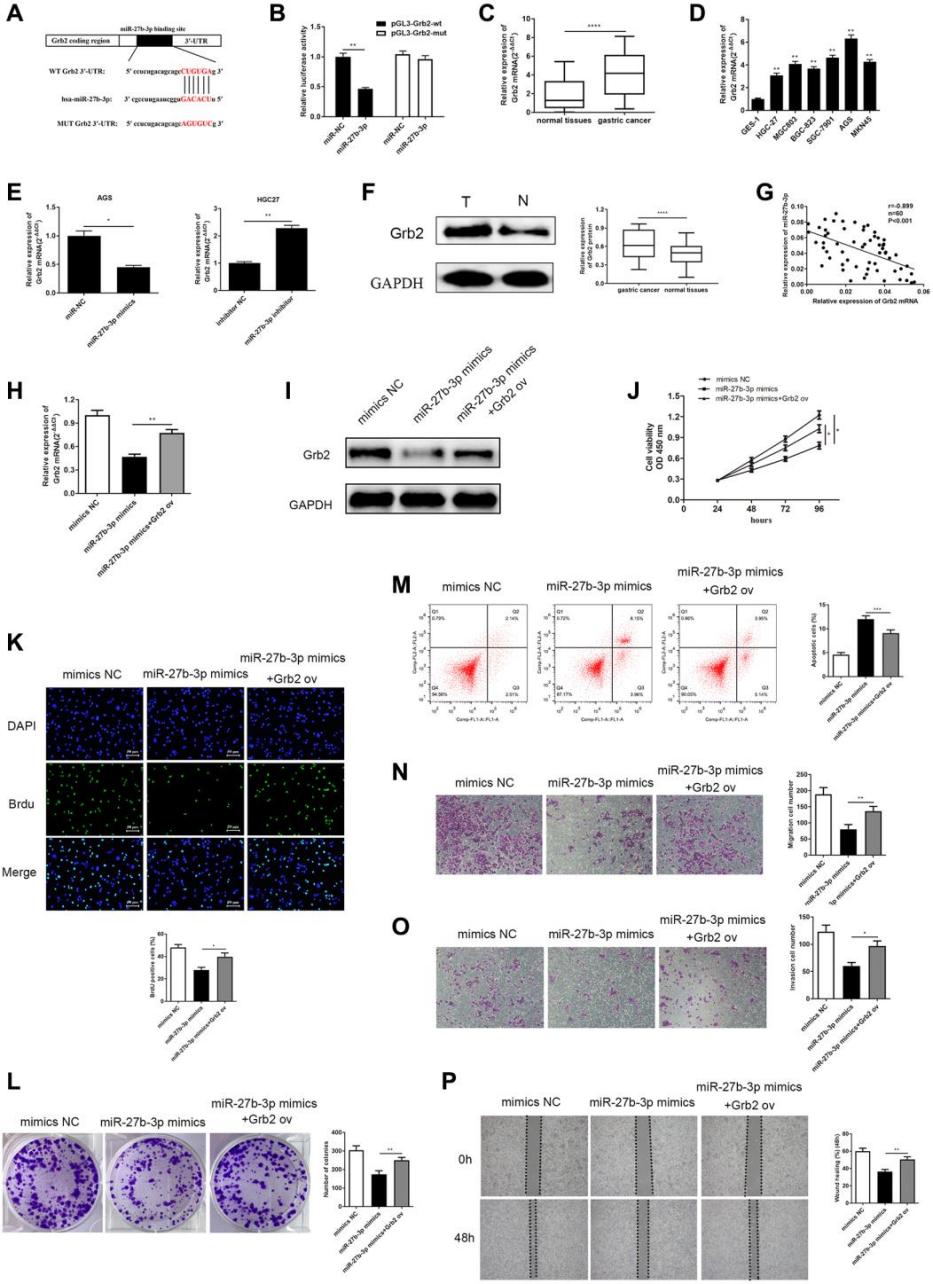
**MiR-27b-3p binds and negatively modulates Grb2, and Grb2 can reverse inhibition of GC cell promotion brought by miR-27b-3p.** (**A**) The complementary sequence between miR-27b-3p and Grb2 3’UTR. (**B**) Luciferase reporter assay verifies a direct relationship between miR-27b-3p and Grb2 (*P* = 0.0041). (**C**) Across 60 GC tissue samples, Grb2 levels were higher compared to adjacent cancer (*P* < 0.0001). (**D**) Grb2 levels in GC cell lines is higher than that in gastric mucosa epithelial cells. AGS had the highest expression, while HGC-27 was relatively low. (**E**) Grb2 mRNA expression was lower in the miR-27b-3p mimics group (*P* = 0.0376) and was higher in the miR-27b-3p inhibitor group (*P* = 0.0034). (**F**) Grb2 expression in GC and adjacent tissues was detected through western blot (*P* < 0.0001). (**G**) There is a negative correlation between Grb2 and miR-27b-3p expression. (**H**–**I**) Up-regulation of Grb2 mRNA and protein levels were determined using qRT-PCR and WB after Grb2 ov in the miR-27b-3p mimics group (*P* = 0.0019). (**J**–**K**) CCK-8 and BrdU assays (*P* = 0.0128) detected GC cell viability and proliferation ability, respectively. Up-regulated Grb2 mRNA levels were positively associated with GC cell growth. (**L**) Colony formation assay detected the numbers of colony formation (*P* = 0.0075). (**M**) GC cell apoptosis was inhibited upon overexpression of Grb2, as examined by flow cytometry assay (*P* = 0.0009). (**N**–**O**) Accelerated cell migration (*P* = 0.0089) and invasion (*P* = 0.0125) abilities were found by transwell assays brought on by increased Grb2 levels. (**P**) Wound healing assay was utilized to discover GC cell metastasis, which indicated that high Grb2 levels promote GC cell metastasis (*P* = 0.0017).

We applied bioinformatics tools to predict the downstream target genes of miR-27b-3p. We utilized the Starbase, miRDB, DIANA, TarBase and TargetScan. Cluster analysis was conducted on downstream target genes that appeared in at least three databases at the same time. The results demonstrated that miR-27b-3p is able to bind to 3′UTR of Grb2 mRNA (Figure 5A). The following dual luciferase assay validated the direct interaction between miR-27b-3p and Grb2. Furthermore, miR-27b-3p is able to markedly reduce luciferase activity compared to the control group in GC cells that are transfected with pGL3-Grb2-wt plasmid, while luciferase activity in GC cells that were transfected with pGL3- Grb2-mut demonstrated no change (Figure 5B).

In order to assess the clinical relevance between miR-27b-3p and Grb2, qRT-PCR was conducted to detect Grb2 mRNA levels both in GC patient tissues and GC cell lines. The expression levels of Grb2 mRNA were significantly up-regulated compared to normal (Figure 5C, 5D). In AGS cells transfected with miR-27b-3p mimics, Grb2 mRNA levels were markedly reduced, however, we observed increasing Grb2 mRNA levels in HGC-27 cells transfected with the miR-27b-3p inhibitor (Figure 5E). Western blot was utilized to determine Grb2 protein levels in GC tissues, and Grb2 levels were higher than adjacent normal tissues (Figure 5F). Pearson’s correlation analysis revealed a negative correlation between Grb2 and miR-27b-3p expression (Figure 5G). From this evidence, we determined that miR-27b-3p directly binds to Grb2 and their expression levels have a negative relationship.

### Grb2 altered miR-27b-3p effects on GC cells, facilitating GC cell promotion

After validating the tumor-restraining effects of miR-27b-3p, and its negative correlation with Grb2 expression, we further assessed their functional relationship in GC cells. We established three groups, including (1) AGS cells transfected with miR-27b-3p mimics, (2) AGS cells co-transfected with miR-27b-3p mimics and Grb2 ov vector and (3) AGS cells transfected with mimics NC vector. As expected, Grb2 mRNA and protein levels were significantly increased upon addition of Grb2 ov vector compared to miR-27b-3p mimics group (Figure 5H, 5I).

Subsequently, cell viability and colony formation abilities were assessed using the CCK-8, BrdU and colony formation assay (Figure 5J–5L). Results from these experiments demonstrated that miR-27b-3p mimics are able to inhibit GC cell viability and colony formation, while Grb2 ov counteracts miR-27b-3p-induced tumor suppressive effects on GC cells. Cell apoptosis, migration and invasion abilities were evaluated using flow cytometry assay (Figure 5M), transwell assays (Figure 5N–5O) and wound healing assay (Figure 5P). MiR-27b-3p mimics induced GC cell apoptosis, and inhibited GC cell migration and invasion. It is clear to see that Grb2 ov neutralized these effects, thereby preventing GC cells apoptosis and accelerating GC cells metastasis. Taken together, Grb2 altered inhibition of GC promotion induced by miR-27b-3p, suggesting that Grb2 may act as a GC indicator.

### Grb2 knockdown attenuated the PAX4 ov-induced effect on GC cell progression

**Figure 6:**
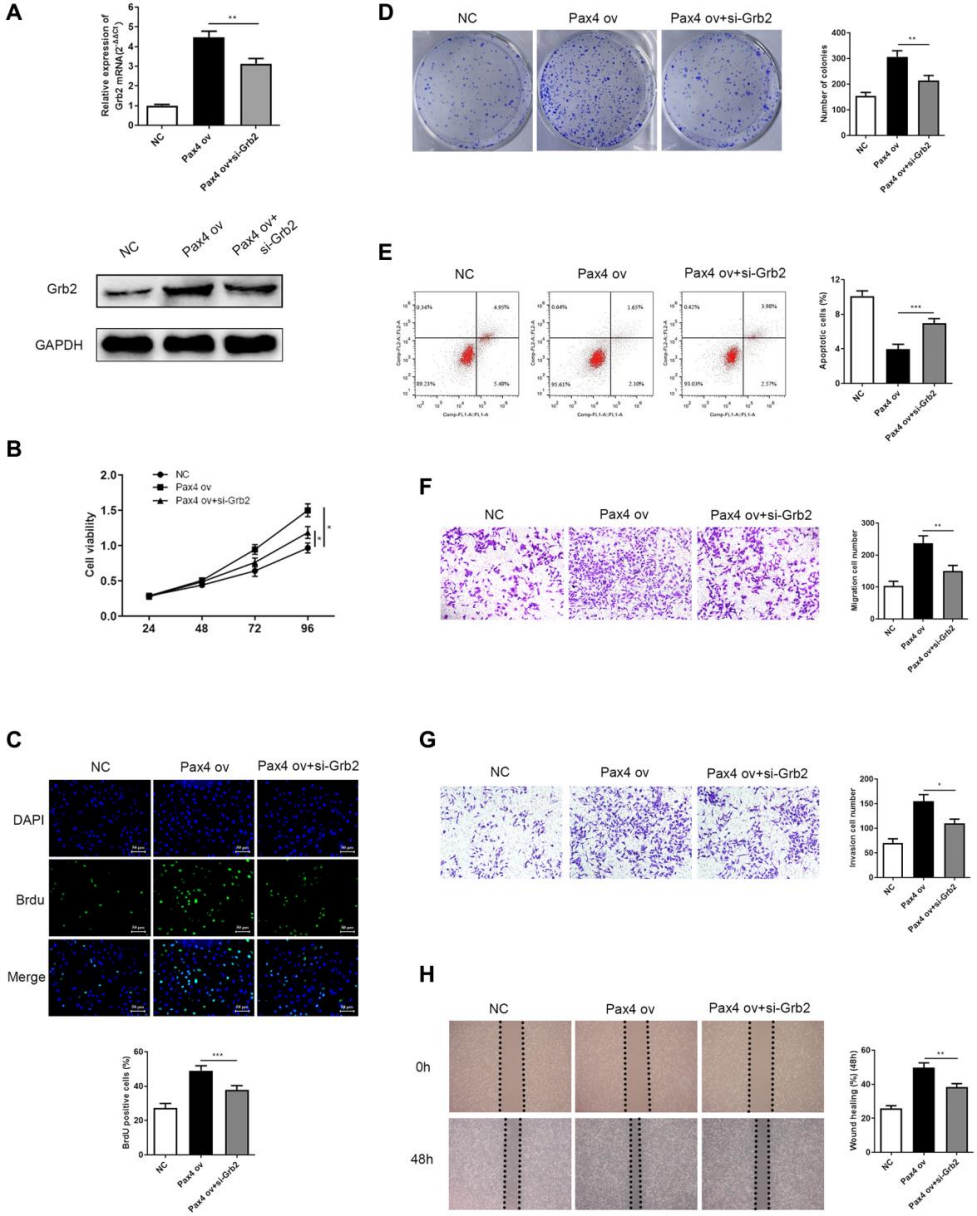
**Grb2 knockdown can attenuate PAX4 ov-induced effect on GC cell progression.** (**A**) Lower Grb2 levels were determined via qRT-PCR and western blot after adding si-Grb2 to the PAX4 ov group (*P* = 0.0025). (**B**–**C**) Grb2 knockdown weakened GC cell proliferation induced by PAX4 ov, through CCK-8 and BrdU assays (*P* = 0.0009). (**D**) Grb2 knockdown decreased PAX4-ov-induced numbers of colony formation, via the colony formation assay (*P* = 0.0013). (**E**) GC cell apoptosis was elevated upon addition of si-Grb2 to the PAX4 ov group (*P* = 0.0004). (**F**–**G**) Reduced cell migration (*P* = 0.0059) and invasion (*P* = 0.0121) was identified after adding si-Grb2 to PAX4 ov group by transwell assays. (**H**) Grb2 knockdown declined migration induced by PAX4 ov by wound healing assay (*P* = 0.0060).

Next, we explored the effect of Grb2 knockdown on PAX4-induced function in GC cells. Firstly, the mRNA and protein expression of Grb2 were determined in NC, PAX4 ov, PAX4 ov+si-Grb2 groups by qRT-PCR and Western Blot. Results indicated significantly lower Grb2 levels upon addition of si-Grb2 to PAX4 ov group (Figure 6A). CCK-8, BrdU and colony formation assays were utilized to demonstrate the effect of Grb2 knockdown on PAX4-induced GC cell proliferation and colony formation ability. The results indicated that down-regulation of Grb2 is able to significantly inhibit GC cell proliferation and colony formation, which is activated by PAX4 ov (Figure 6B–6D). Flow cytometry results prove that adding si-Grb2 increased GC cell apoptosis, which is weakened by PAX4 ov (Figure 6E). The transwell assays and wound healing assays in revealed that Grb2 knockdown was able to reserve the promotion of GC cell migration and invasion mediated by PAX4 ov (Figure 6F–6H). Taken together, Grb2 knockdown attenuates the PAX4 ov-induced effect on GC cell progression.

### PAX4 activated Ras-ERK pathway via regulating miR-27b-3p /Grb2

**Figure 7:**
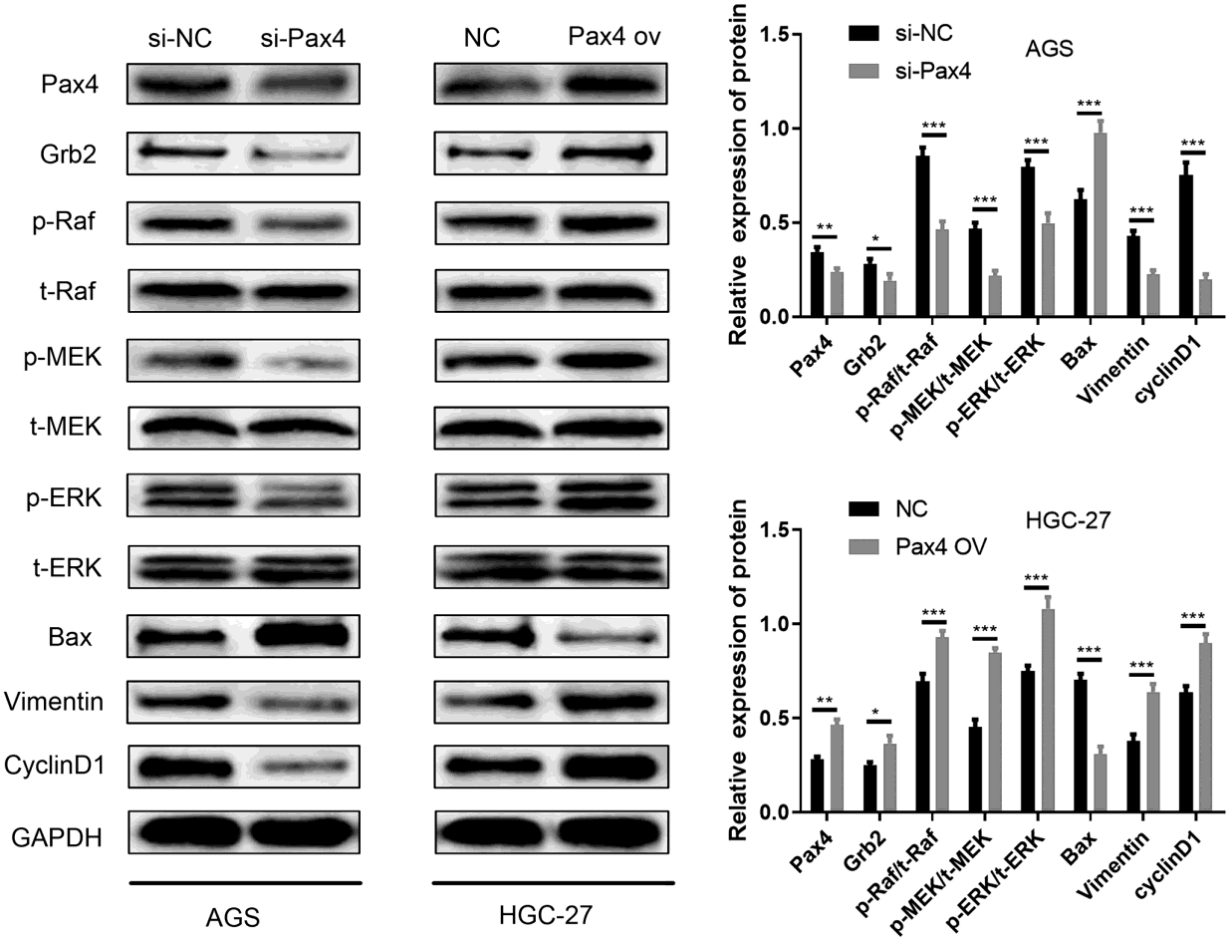
**PAX4/miR-27b-3p/Grb2 regulatory loop activates the Ras-ERK pathway.** Ras-ERK associated proteins (p-Raf, p-MEK, p-ERK, t-Raf, t-MEK, t-ERK), EMT pathway protein (Vimentin) and cyclin pathway-related protein (cyclinD1) and Bax apoptotic protein were determined using western blot.

The Ras-ERK pathway is one of the most well-studied signaling pathways. This pathway is involved in a variety of physiological and pathological processes, including cell proliferation, growth, development, differentiation, and malignant transformation of cells. The Ras-ERK pathway is also known to be significant in tumorigenesis and development. MiR-27-3p and Grb2 have been reported to be associated with the Ras-ERK pathway across diverse cancers [30–34]. As mentioned above, we have already validated that PAX4 influences GC cell promotion by modulating the miR-27b-3p/Grb2 axis. Therefore, the PAX4/miR-27b-3p/Grb2 regulatory loop may be associated with the Ras-ERK pathway. Related biomarker proteins (p-Raf, p-MEK, p-ERK) were determined using western blot. In the si-PAX4 group, levels of p-Raf, p-MEK, p-ERK were down-regulated compared to the si-NC group while total Raf (t-Raf), total ERK (t-ERK), total MEK (t-MEK) levels were not significantly different (Figure 7). Inhibition of the Ras-ERK pathway further resulted in increased Bax apoptotic protein, decreased vimentin (EMT pathway protein) and decreased cyclinD1 (cyclin). However, the group with PAX4 overexpression showed the opposite. Overexpressed p-Raf, p-MEK, p-ERK levels were observed, while the total Raf (t-Raf), total ERK (t-ERK), total MEK (t-MEK) levels remained the same. Lower levels of the Bax apoptotic protein and higher levels of Vimentin (EMT pathway protein) and cyclinD1 (cyclin) were provided, in comparison to the control group (Figure 7). The results supported that PAX4/miR-27b-3p /Grb2 regulatory loop activates the Ras-ERK pathway, which is associated with GC cell progression.

## DISCUSSION

PAX4, encoded by the Paired box4 (PAX4) gene, is a member of the paired homologous domain transcription factor PAX family. The DNA binding domain of PAX4 is formed via the homeo-like domain of the paired structural domains, which belong to the fourth subfamily of the PAX family. The PAX family of transcription factors are important regulators of tissue development and cell differentiation, which are known to be involved in regulating cell proliferation, apoptosis and migration [35]. Abnormal expression of PAX genes are known to cause congenital disorders. For example, abnormal expression of PAX1 is able to inhibit malignant phenotypes under oncogenic stress conditions, while PAX3 mutation can cause neurodevelopmental diseases, disturb mRNA transcription [36, 37]. PAX2 can cause renal anomaly development by regulating expression of TBX1 [38]. Studies of PAX4 have mainly focused on the development of pancreatic β, δ-cells and metabolic diseases, though it has been reported PAX4 expression is dysregulated in human epithelial cancers [39]. PAX4 is located on chromosome 7q32 [11]. This site has been identified as a frequent target of chromosomal alterations in human tumors. Deletions and translocation breakpoints at chromosome 7q32 have been frequently observed, especially in lymphoid malignancies [40–42]. A previous study demonstrated that PAX4 is aberrantly expressed in primary lymphomas. and that these expression levels are caused by aberrant DNA demethylation of the promoter region of *PAX4* [23]. However, these reports were based only on the relationship between PAX4 expression and cancer malignant phenotypes. Hence, the underlying mechanisms were not investigated. Little research has focused on the detailed targets of PAX4. Therefore, we can identify possible regulatory mechanism of abnormal PAX4 expression in GC. In our previous study, using qRT-PCR, WB and IHC methods, we identified that the expression of PAX4 in GC tissues was significantly higher compared to adjacent tissues, which suggests that PAX4 likely plays a role in promoting the occurrence and development of GC.

In recent years, an increasing number of studies have demonstrated a mutual regulatory relationship between transcription factors and miRNAs, which bind to the upstream promoter region of corresponding miRNA genes and have an effect on miRNA transcription [43, 44]. Singh SK et al. identified that in the differentiation and self-renewal of mouse embryonic stem cells, the transcription factor REST binds to specific binding sites that correspond to specific miRNA genes. Additionally, when REST is added to mouse embryonic stem cells, the expression levels of corresponding miRNAs become reduced. Reducing REST expression increase expression of these miRNAs [45, 46]. Bioinformatics tools predict that there are certain binding sites between PAX4 and miR-27b-3p promoter region, which has been confirmed by ChIP and dual luciferase assays. Thus, we discovered that PAX4 directly interacts with miR-27b-3p in GC cells.

Next, we used bioinformatics tools to predict downstream target genes of miR-27b-3p, the results of which showed that miR-27b-3p is able to combine with the 3′UTR of the Grb2 mRNA. Furthermore, overexpressed miR-27b-3p significantly lowered Grb2 expression in GC cells, while inhibition of miR-27b-3p is able to significantly raise Grb2 expression.

Earlier studies have identified that GRB2, which consists of two SH3 domains and one Src homology 2 (SH2) domain, is a receptor tyrosine kinase-interacting protein that is the key to defining RTK signaling [47]. The interaction between GRB2 and SOS, and the recruitment of the GRB2-SOS complex to the plasma membrane in order to activate the MAPK protein kinase cascade is an important function of GRB2 in cells [24]. Consequently, GRB2 is able to connect EGFR and downstream Ras/Erk signaling molecules [47]. GRB2 promotes tumor cell proliferation and inhibits apoptosis by activating the Ras/Erk pathway, while inhibiting the GRB2/Ras/Erk pathway can limit tumor development by promoting apoptosis [48–50]. The Ras-ERK pathway participates in a variety of physiological and pathological processes, including cell proliferation, growth, development, differentiation, and malignant transformation of cells, and is of great significance in tumorigenesis and development [51]. We conducted western blot to validate the activation of the Ras-ERK pathway brought by PAX4/miR-27b-3p/Grb2.

Therefore, we concluded that the abnormal expression of transcription factor PAX4 regulates miR-27b-3p expression, which causes higher Grb2 levels, and results in Ras-ERK signaling pathway abnormal activation. Overall, this initiates Ras-ERK signaling pathway downstream target gene transcription, promotes GC cell proliferation and inhibits GC cell apoptosis. PAX4/ miR-27b-3p/Grb2 regulatory loop plays a significant role in GC tumor progression, which indicates that potential therapy biomarkers need to be further investigated. This work provides the first clear that PAX4 plays a significant role GC, and will give us more inspiration on tumor target therapy.

## CONCLUSIONS

Pax4 is a transcription factor that is highly expressed in GC. Transcription factors and downstream molecules of DNA promoter region will combine, promote or inhibit the DNA transcription of downstream. Our prediction demonstrated that Pax4 may combine with the promoter area of miR-27b-3p, which prevents transcription of miR-27b-3p and leads to the increasing expression of the downstream molecule Grb2. Overall, this causes abnormal Ras-ERK signaling pathway activation.

## References

[1] Bray F, Ferlay J, Soerjomataram I, Siegel RL, Torre LA, Jemal A. Global cancer statistics 2018: GLOBOCAN estimates of incidence and mortality worldwide for 36 cancers in 185 countries. CA Cancer J Clin. 2018; 68:394–424. 10.3322/caac.21492 30207593

[2] Epplein M, Butt J, Zhang Y, Hendrix LH, Abnet CC, Murphy G, Zheng W, Shu XO, Tsugane S, Qiao YL, Taylor PR, Shimazu T, Yoo KY, et al. Validation of a Blood Biomarker for Identification of Individuals at High Risk for Gastric Cancer. Cancer Epidemiol Biomarkers Prev. 2018; 27:1472–79. 10.1158/1055-9965.EPI-18-0582 30158280 PMC6279536

[3] Thrift AP, El-Serag HB. Burden of Gastric Cancer. Clin Gastroenterol Hepatol. 2020; 18:534–42. 10.1016/j.cgh.2019.07.045 31362118 PMC8859863

[4] Hong S, Won YJ, Park YR, Jung KW, Kong HJ, Lee ES, and Community of Population-Based Regional Cancer Registries. Cancer Statistics in Korea: Incidence, Mortality, Survival, and Prevalence in 2017. Cancer Res Treat. 2020; 52:335–50. 10.4143/crt.2020.206 32178489 PMC7176962

[5] Rawla P, Barsouk A. Epidemiology of gastric cancer: global trends, risk factors and prevention. Prz Gastroenterol. 2019; 14:26–38. 10.5114/pg.2018.80001 30944675 PMC6444111

[6] Sisik A, Kaya M, Bas G, Basak F, Alimoglu O. CEA and CA 19-9 are still valuable markers for the prognosis of colorectal and gastric cancer patients. Asian Pac J Cancer Prev. 2013; 14:4289–94. 10.7314/apjcp.2013.14.7.4289 23991991

[7] Qing Y, Li Q, Ren T, Xia W, Peng Y, Liu GL, Luo H, Yang YX, Dai XY, Zhou SF, Wang D. Upregulation of PD-L1 and APE1 is associated with tumorigenesis and poor prognosis of gastric cancer. Drug Des Devel Ther. 2015; 9:901–09. 10.2147/DDDT.S75152 25733810 PMC4338255

[8] Yuan HL, Wang T, Zhang KH. MicroRNAs as potential biomarkers for diagnosis, therapy and prognosis of gastric cancer. Onco Targets Ther. 2018; 11:3891–900. 10.2147/OTT.S156921 30013369 PMC6039071

[9] Ghebeh H, Tulbah A, Mohammed S, Elkum N, Bin Amer SM, Al-Tweigeri T, Dermime S. Expression of B7-H1 in breast cancer patients is strongly associated with high proliferative Ki-67-expressing tumor cells. Int J Cancer. 2007; 121:751–58. 10.1002/ijc.22703 17415709

[10] Durães C, Almeida GM, Seruca R, Oliveira C, Carneiro F. Biomarkers for gastric cancer: prognostic, predictive or targets of therapy? Virchows Arch. 2014; 464:367–78. 10.1007/s00428-013-1533-y 24487788

[11] Yoshiura KI, Kubota T, Soejima H, Tamura T, Izumikawa Y, Niikawa N, Jinno Y. A comparison of GC content and the proportion of Alu/KpnI-repetitive sequences in a single dark- and light-band region from a human chromosome. Genomics. 1994; 20:243–48. 10.1006/geno.1994.1160 8020971

[12] Fujitani Y, Kajimoto Y, Yasuda T, Matsuoka TA, Kaneto H, Umayahara Y, Fujita N, Watada H, Miyazaki JI, Yamasaki Y, Hori M. Identification of a portable repression domain and an E1A-responsive activation domain in Pax4: a possible role of Pax4 as a transcriptional repressor in the pancreas. Mol Cell Biol. 1999; 19:8281–91. 10.1128/MCB.19.12.8281 10567553 PMC84912

[13] Lang D, Powell SK, Plummer RS, Young KP, Ruggeri BA. PAX genes: roles in development, pathophysiology, and cancer. Biochem Pharmacol. 2007; 73:1–14. 10.1016/j.bcp.2006.06.024 16904651

[14] Brun T, Gauthier BR. A focus on the role of Pax4 in mature pancreatic islet beta-cell expansion and survival in health and disease. J Mol Endocrinol. 2008; 40:37–45. 10.1677/JME-07-0134 18234907

[15] Sujjitjoon J, Kooptiwut S, Chongjaroen N, Semprasert N, Hanchang W, Chanprasert K, Tangjittipokin W, Yenchitsomanus PT, Plengvidhya N. PAX4 R192H and P321H polymorphisms in type 2 diabetes and their functional defects. J Hum Genet. 2016; 61:943–49. 10.1038/jhg.2016.80 27334367

[16] Chapla A, Mruthyunjaya MD, Asha HS, Varghese D, Varshney M, Vasan SK, Venkatesan P, Nair V, Mathai S, Paul TV, Thomas N. Maturity onset diabetes of the young in India - a distinctive mutation pattern identified through targeted next-generation sequencing. Clin Endocrinol (Oxf). 2015; 82:533–42. 10.1111/cen.12541 25041077

[17] Lorenzo PI, Cobo-Vuilleumier N, Gauthier BR. Therapeutic potential of pancreatic PAX4-regulated pathways in treating diabetes mellitus. Curr Opin Pharmacol. 2018; 43:1–10. 10.1016/j.coph.2018.07.004 30048825

[18] Lorenzo PI, Juárez-Vicente F, Cobo-Vuilleumier N, García-Domínguez M, Gauthier BR. The Diabetes-Linked Transcription Factor PAX4: From Gene to Functional Consequences. Genes (Basel). 2017; 8:101. 10.3390/genes8030101 28282933 PMC5368705

[19] Schaner ME, Ross DT, Ciaravino G, Sorlie T, Troyanskaya O, Diehn M, Wang YC, Duran GE, Sikic TL, Caldeira S, Skomedal H, Tu IP, Hernandez-Boussard T, et al. Gene expression patterns in ovarian carcinomas. Mol Biol Cell. 2003; 14:4376–86. 10.1091/mbc.e03-05-0279 12960427 PMC266758

[20] Fabbro D, Di Loreto C, Beltrami CA, Belfiore A, Di Lauro R, Damante G. Expression of thyroid-specific transcription factors TTF-1 and PAX-8 in human thyroid neoplasms. Cancer Res. 1994; 54:4744–49. 8062273

[21] Li CG, Eccles MR. PAX Genes in Cancer; Friends or Foes? Front Genet. 2012; 3:6. 10.3389/fgene.2012.00006 22303411 PMC3269002

[22] Zhang J, Qin X, Sun Q, Guo H, Wu X, Xie F, Xu Q, Yan M, Liu J, Han Z, Chen W. Transcriptional control of PAX4-regulated miR-144/451 modulates metastasis by suppressing ADAMs expression. Oncogene. 2015; 34:3283–95. 10.1038/onc.2014.259 25151965

[23] Li Y, Nagai H, Ohno T, Ohashi H, Murohara T, Saito H, Kinoshita T. Aberrant DNA demethylation in promoter region and aberrant expression of mRNA of PAX4 gene in hematologic malignancies. Leuk Res. 2006; 30:1547–53. 10.1016/j.leukres.2006.04.001 16701883

[24] Brun T, Duhamel DL, Hu He KH, Wollheim CB, Gauthier BR. The transcription factor PAX4 acts as a survival gene in INS-1E insulinoma cells. Oncogene. 2007; 26:4261–71. 10.1038/sj.onc.1210205 17260022

[25] Zhang J, Hua X, Qi N, Han G, Yu J, Yu Y, Wei X, Li H, Chen X, Leng C, Liu Q, Lu Y, Li Y. MiR-27b suppresses epithelial-mesenchymal transition and chemoresistance in lung cancer by targeting Snail1. Life Sci. 2020; 254:117238. 10.1016/j.lfs.2019.117238 31887300

[26] Ahmed Z, Lin CC, Suen KM, Melo FA, Levitt JA, Suhling K, Ladbury JE. Grb2 controls phosphorylation of FGFR2 by inhibiting receptor kinase and Shp2 phosphatase activity. J Cell Biol. 2013; 200:493–504. 10.1083/jcb.201204106 23420874 PMC3575544

[27] Ma Q, Song J, Ma H, Gao K, Yang Y, He N. Synergistic anticancer effect of Grb2 and ITGA1 on cancer cells highly expressing Grb2 through suppressing ERK phosphorylation. Int J Clin Exp Pathol. 2019; 12:182–89. 31933732 PMC6944030

[28] Daly RJ, Binder MD, Sutherland RL. Overexpression of the Grb2 gene in human breast cancer cell lines. Oncogene. 1994; 9:2723–27. 8058337

[29] Li LY, Li EM, Wu ZY, Cao HH, Shen JH, Xu XE, Chen B, Wu JY, Xu LY. Overexpression of GRB2 is correlated with lymph node metastasis and poor prognosis in esophageal squamous cell carcinoma. Int J Clin Exp Pathol. 2014; 7:3132–40. 25031732 PMC4097250

[30] Kim H, Yang JM, Jin Y, Jheon S, Kim K, Lee CT, Chung JH, Paik JH. MicroRNA expression profiles and clinicopathological implications in lung adenocarcinoma according to EGFR, KRAS, and ALK status. Oncotarget. 2017; 8:8484–98. 10.18632/oncotarget.14298 28035073 PMC5352416

[31] Chen D, Si W, Shen J, Du C, Lou W, Bao C, Zheng H, Pan J, Zhong G, Xu L, Fu P, Fan W. miR-27b-3p inhibits proliferation and potentially reverses multi-chemoresistance by targeting CBLB/GRB2 in breast cancer cells. Cell Death Dis. 2018; 9:188. 10.1038/s41419-017-0211-4 29416005 PMC5833695

[32] Yang F, Zhang W, Shen Y, Guan X. Identification of dysregulated microRNAs in triple-negative breast cancer (review). Int J Oncol. 2015; 46:927–32. 10.3892/ijo.2015.2821 25571912

[33] Jiang W, Wei K, Pan C, Li H, Cao J, Han X, Tang Y, Zhu S, Yuan W, He Y, Xia Y, Chen L, Chen Y. MicroRNA-1258 suppresses tumour progression via GRB2/Ras/Erk pathway in non-small-cell lung cancer. Cell Prolif. 2018; 51:e12502. 10.1111/cpr.12502 30069987 PMC6528891

[34] Skolnik EY, Batzer A, Li N, Lee CH, Lowenstein E, Mohammadi M, Margolis B, Schlessinger J. The function of GRB2 in linking the insulin receptor to Ras signaling pathways. Science. 1993; 260:1953–55. 10.1126/science.8316835 8316835

[35] Miller DJ, Hayward DC, Reece-Hoyes JS, Scholten I, Catmull J, Gehring WJ, Callaerts P, Larsen JE, Ball EE. Pax gene diversity in the basal cnidarian Acropora millepora (Cnidaria, Anthozoa): implications for the evolution of the Pax gene family. Proc Natl Acad Sci U S A. 2000; 97:4475–80. 10.1073/pnas.97.9.4475 10781047 PMC18259

[36] Su PH, Lai HC, Huang RL, Chen LY, Wang YC, Wu TI, Chan MWY, Liao CC, Chen CW, Lin WY, Chang CC. Paired Box-1 (PAX1) Activates Multiple Phosphatases and Inhibits Kinase Cascades in Cervical Cancer. Sci Rep. 2019; 9:9195. 10.1038/s41598-019-45477-5 31235851 PMC6591413

[37] Arasu A, Murugan S, Essa MM, Velusamy T, Guillemin GJ. PAX3: A Molecule with Oncogenic or Tumor Suppressor Function Is Involved in Cancer. BioMed Research International. 2018; 2018:1095459.

[38] Jiang H, Li L, Yang H, Bai Y, Jiang H, Li Y. Pax2 may play a role in kidney development by regulating the expression of TBX1. Mol Biol Rep. 2014; 41:7491–98. 10.1007/s11033-014-3639-y 25106525

[39] Napolitano T, Avolio F, Courtney M, Vieira A, Druelle N, Ben-Othman N, Hadzic B, Navarro S, Collombat P. Pax4 acts as a key player in pancreas development and plasticity. Semin Cell Dev Biol. 2015; 44:107–14. 10.1016/j.semcdb.2015.08.013 26319183

[40] Offit K, Wong G, Filippa DA, Tao Y, Chaganti RS. Cytogenetic analysis of 434 consecutively ascertained specimens of non-Hodgkin’s lymphoma: clinical correlations. Blood. 1991; 77:1508–15. 2009370

[41] Cigudosa JC, Parsa NZ, Louie DC, Filippa DA, Jhanwar SC, Johansson B, Mitelman F, Chaganti RS. Cytogenetic analysis of 363 consecutively ascertained diffuse large B-cell lymphomas. Genes Chromosomes Cancer. 1999; 25:123–33. 10337996

[42] Jerkeman M, Johansson B, Akerman M, Cavallin-Ståhl E, Kristoffersson U, Mitelman F. Prognostic implications of cytogenetic aberrations in diffuse large B-cell lymphomas. Eur J Haematol. 1999; 62:184–90. 10.1111/j.1600-0609.1999.tb01742.x 10089896

[43] Wu E, Thivierge C, Flamand M, Mathonnet G, Vashisht AA, Wohlschlegel J, Fabian MR, Sonenberg N, Duchaine TF. Pervasive and cooperative deadenylation of 3′UTRs by embryonic microRNA families. Mol Cell. 2010; 40:558–70. 10.1016/j.molcel.2010.11.003 21095586 PMC3698950

[44] O'Donnell KA, Wentzel EA, Zeller KI, Dang CV, Mendell JT. c-Myc-regulated microRNAs modulate E2F1 expression. Nature. 2005; 435:839–43. 10.1038/nature03677 15944709

[45] Singh SK, Kagalwala MN, Parker-Thornburg J, Adams H, Majumder S. REST maintains self-renewal and pluripotency of embryonic stem cells. Nature. 2008; 453:223–27. 10.1038/nature06863 18362916 PMC2830094

[46] Gao Z, Ding P, Hsieh J. Profiling of REST-Dependent microRNAs Reveals Dynamic Modes of Expression. Front Neurosci. 2012; 6:67. 10.3389/fnins.2012.00067 22590451 PMC3349273

[47] Lowenstein EJ, Daly RJ, Batzer AG, Li W, Margolis B, Lammers R, Ullrich A, Skolnik EY, Bar-Sagi D, Schlessinger J. The SH2 and SH3 domain-containing protein GRB2 links receptor tyrosine kinases to ras signaling. Cell. 1992; 70:431–42. 10.1016/0092-8674(92)90167-b 1322798

[48] Gril B, Vidal M, Assayag F, Poupon MF, Liu WQ, Garbay C. Grb2-SH3 ligand inhibits the growth of HER2+ cancer cells and has antitumor effects in human cancer xenografts alone and in combination with docetaxel. Int J Cancer. 2007; 121:407–15. 10.1002/ijc.22674 17372910 PMC2755772

[49] Li D, Wu LJ, Tashiro S, Onodera S, Ikejima T. Oridonin-induced A431 cell apoptosis partially through blockage of the Ras/Raf/ERK signal pathway. J Pharmacol Sci. 2007; 103:56–66. 10.1254/jphs.fpj06016x 17251686

[50] Shan X, Miao Y, Fan R, Song C, Wu G, Wan Z, Zhu J, Sun G, Zha W, Mu X, Zhou G, Chen Y. Suppression of Grb2 expression improved hepatic steatosis, oxidative stress, and apoptosis induced by palmitic acid *in vitro* partly through insulin signaling alteration. In Vitro Cell Dev Biol Anim. 2013; 49:576–82. 10.1007/s11626-013-9646-9 23771793

[51] Mendoza MC, Er EE, Blenis J. The Ras-ERK and PI3K-mTOR pathways: cross-talk and compensation. Trends Biochem Sci. 2011; 36:320–28. 10.1016/j.tibs.2011.03.006 21531565 PMC3112285

